# Dr. Samuel Gross

**Published:** 1882-04-20

**Authors:** 


DR. SAMUEL GROSS, the distinguished Surgeon, has resigned his Professorship in Jefferson Medical College.

